# Dr. Chapin Harris

**Published:** 1861-01

**Authors:** 


CHAPIN HARRIS.—Dr. Chapin Harris expired at Baltimore on the twenty-ninth of September, 1860, in the fifty-sixth year of his age. His illness was severe and protracted, confining him to his house for a number of months. In July his health gradually improved, and by the advice of some friends he was induced to visit Atlantic City, in the hope that sea-air and sea-bathing might prove serviceable to his exhausted system; but after a brief sojourn he was compelled to return, and soon afterward died in the bosom of his family.

Dr. Harris was the founder of the American Dental College at Baltimore, the first institution of the kind in the world; and he was the author of a Dictionary of Dental Surgery, a Treatise on Medical Terminology, the American Journal of Dental Science, and the Principles and Practices of Dental Surgery; a work which has passed through numerous editions, and which, together with his other literary labors, has made his name known far and wide both at home and abroad. He wielded at once a prolific and an able pen. His practice for many years was large and lucrative, and he was withal an excellent teacher. He occupied up to the time of his death the chair of the Principles and Practice of Dental Surgery. He was a graduate of medicine, and a member of the American Medical Association. His moral character was distinguished by great excellence and purity. He was tall in person, kind and affable in his manners, and universally beloved for his many good qualities.

Although not strictly of the medical profession, we pay this brief tribute to the memory of Professor Harris the more willingly because of his many virtues, and because of the great zeal and interest which he manifested in the cause of medical and dental science. At the time of his decease he deservedly occupied the very highest position in his particular department of science.

